# Puumala and Tula Virus Differ in Replication Kinetics and Innate Immune Stimulation in Human Endothelial Cells and Macrophages

**DOI:** 10.3390/v11090855

**Published:** 2019-09-14

**Authors:** Daniel Bourquain, Clemens Bodenstein, Stefanie Schürer, Lars Schaade

**Affiliations:** Centre for Biological Threats and Special Pathogens, Robert Koch Institute, 13353 Berlin, Germany; bodensteinc@rki.de (C.B.); schuerers@rki.de (S.S.); schaadel@rki.de (L.S.)

**Keywords:** hantavirus, orthohantavirus, Puumala virus, Tula virus

## Abstract

Old world hantaviruses cause hemorrhagic fever with renal syndrome (HFRS) upon zoonotic transmission to humans. In Europe, the Puumala virus (PUUV) is the main causative agent of HFRS. Tula virus (TULV) is also widely distributed in Europe, but there is little knowledge about the pathogenicity of TULV for humans, as reported cases are rare. We studied the replication of TULV in different cell types in comparison to the pathogenic PUUV and analyzed differences in stimulation of innate immunity. While both viruses replicated to a similar extent in interferon (IFN)-deficient Vero E6 cells, TULV replication in human lung epithelial (A549) cells was slower and less efficient when compared to PUUV. In contrast to PUUV, no replication of TULV could be detected in human microvascular endothelial cells and in macrophages. While a strong innate immune response towards PUUV infection was evident at 48 h post infection, TULV infection triggered only a weak IFN response late after infection of A549 cells. Using appropriate in vitro cell culture models for the orthohantavirus infection, we could demonstrate major differences in host cell tropism, replication kinetics, and innate immune induction between pathogenic PUUV and the presumably non- or low-pathogenic TULV that are not observed in Vero E6 cells and may contribute to differences in virulence.

## 1. Introduction

Hantaviruses are three segmented negative-stranded RNA viruses which are harbored by small mammals. They form the genus *Orthohantavirus* within the *Hantaviridae* family of the order *Bunyavirales.* Upon zoonotic transmission to humans via aerosols, they cause a disease known as hemorrhagic fever with renal syndrome (HFRS) in the old world and hantavirus cardiopulmonary syndrome (HCPS) in the new world [1]. Hantavirus-associated diseases in Europe are mainly caused by infections with Puumala virus (PUUV) carried by *Myodes* voles and to a lesser extent by Dobrava-Belgrade virus (DOBV) carried by different *Apodemus* species [2]. While PUUV causes mainly a mild form of HFRS, also known as nephropathia epidemica [3], DOBV infections tend to be more severe [2,4]. A third hantavirus, Tula virus (TULV), is carried by *Microtus* voles which are widely distributed in Europe [2,5,6,7]. TULV infection in humans has been serologically documented in blood donors in the Czech Republic [8] and in German forestry workers, a potential risk group for hantavirus infections [9]. There is little knowledge about the pathogenicity of TULV, as reported cases of disease caused by TULV infection are rare, without any fatalities known so far. One HFRS patient from Germany had TULV-specific neutralizing antibodies [10]. In addition, TULV RNA was detected in EDTA blood of an acutely infected, immunocompromised patient in the Czech Republic [11]. Furthermore, TULV infection was detected in a hospitalized patient in France in 2015 [12]. However, as often no differentiation is made between infections by TULV or the closely related PUUV, more cases of human TULV infections may exist which are misdiagnosed as PUUV infections [13].

In human hantavirus infections, a dysregulation of endothelial cell functions—either caused by the infection itself or by an excessive immune response towards the infection—is thought to be the cause of the hantavirus-induced pathologies [14,15]. However, the determinants for the diverse degrees of hantavirus pathogenicity observed in humans are still unclear. Differences in receptor usage may play a role, as pathogenic hantaviruses like PUUV enter cells via β3 integrins while low-pathogenic hantaviruses like TULV utilize β1 integrins for entry, and subversion of the β3 integrin signaling pathway is thought to compromise vascular integrity [15]. Furthermore, differences in entry mechanisms or modulation of the host cell machinery may in turn affect viral replication kinetics and thereby determine hantavirus virulence [15,16]. Differential regulation of the innate immune response is also considered as one of the pathogenicity determinants. Like all viruses, hantaviruses need to prevent early induction of the cellular antiviral interferon (IFN) response in order to replicate successfully in human cells [17,18,19]. Several reports have shown that hantavirus replication is sensitive to IFN and that IFN induction by hantavirus infection differs between viral species (reviewed in [20]). The non-pathogenic prospect hill virus (PHV) has been shown to differ from other hantaviruses in its inability to restrict early type I IFN responses, rendering it unable to replicate in endothelial cells [21,22]. However, while early activation of innate immune responses limits viral replication and thereby the development of hantavirus pathology, a delayed and subsequently exaggerated innate immune response towards uncontrolled viral replication most likely contributes to pathogenicity [16,23,24,25,26]. This suggests that the ability of hantaviruses to modulate innate immunity actually relates to their various degrees of pathogenicity.

In this study, we compared the replication efficiency of the pathogenic PUUV and the non- or low-pathogenic TULV in different cell types and analyzed differences in immune stimulation between these viruses. In human infections, hantaviruses mainly infect endothelial cells and macrophages. As an in vitro model for human endothelial cells, the well-characterized cell line HMEC-1 was used [27], which closely resembles microvascular endothelial cells in regard to many phenotypic characteristics [28,29]. Infection of macrophages was studied in PMA-differentiated THP-1 cells in comparison to peripheral blood mononuclear cell (PBMC)-derived macrophages. Furthermore, infection of lung epithelial cells was studied, which may in vivo represent the first cells to be in contact with the virus after inhalation of hantavirus particles. In this study, we used A549 lung epithelial cells, which have been widely used as an in vitro model system for hantavirus infections, including gene expression profiling [30,31,32,33,34,35]. Our results show that PUUV replicates efficiently in all tested cell types, whereas TULV replication was considerably weaker or even undetectable in all cell types except IFN-deficient Vero E6 cells. While the rapid PUUV replication induced a strong IFN response as early as 48 h post infection (p.i.), no stimulation of innate immune responses by TULV was monitored during the first six days post infection. In case these differences in host cell tropism, replication kinetics, and innate immune stimulation observed in vitro are also present in the more complex in vivo situation, these may contribute to the higher pathogenicity of PUUV in comparison to TULV in the human host.

## 2. Materials and Methods

### 2.1. Virus Cultivation

PUUV (strain Kazan) [36] and TULV (strain Moravia-5302Ma-94) [8] were grown on Vero E6 cells (American Type Culture Collection (ATCC), Manassas, VA, USA; CRL 1586) in T175 cell culture flasks under standard cell culture conditions (Dulbecco’s Modified Eagle’s Medium (DMEM), 5% fetal calf serum (FCS), 37 °C). Cells were infected in 2 mL of DMEM containing 1% FCS for 2 h using a multiplicity of infection (MoI) of 0.005. Subsequently, 28 mL of DMEM medium containing 5% FCS were added and the cells were incubated for 7 d. At 7 d p.i. 20 mL of the virus containing cell culture supernatant were harvested, followed by a centrifugation step to remove cellular debris (1000× *g*, 5 min, 4 °C). The cells were disrupted in the remaining 10 mL of medium by three consecutive freeze/thaw cycles (−20 °C) followed by sonication (Bioruptor Plus, Diagenode SA, Seraing, Belgium; 3 × 30 s, 4 °C, high intensity) and removal of cellular debris via centrifugation (1000× *g*, 5 min, 4 °C). Subsequently, the virus containing supernatants were pooled and viral particles were enriched via sucrose cushion ultracentrifugation (30% sucrose; 175,000× *g*, 1.5 h, 4 °C). Viral stocks were titrated by focus forming unit (FFU) assay and stored in aliquots at −80 °C for further use. Stocks of comparable virus titers of approximately 1 × 10^7^ FFU/mL were used for infection experiments. All virus stocks were tested and found to be negative for mycoplasma and SV5 contamination. Biosafety level 2 facilities were used for virus cultivation and experimental infections.

### 2.2. Cell Lines

A549 human lung carcinoma epithelial cells (ATCC^®^ CCL-185™), HMEC-1 human microvascular endothelial cells (ATCC^®^ CRL-3243), THP-1 human monocytic cells (ATCC^®^ TIB-202™) and Vero E6 green monkey epithelial kidney cells (ATCC CRL-1586) were cultured under standard conditions in 24-well cell culture plates. A549 cells and Vero E6 cells were cultivated in DMEM containing 10% FCS and 2 mM L-Glutamine. HMEC-1 cells were cultivated in MCDB131 medium containing 10% FCS, 10 mM L-Glutamine, 10 ng/mL epidermal growth factor (EGF) and 1 µg/mL Hydrocortisone. THP-1 cells were cultivated in RPMI medium containing 10% FCS and 2 mM L-Glutamine. For differentiation into a macrophage-like phenotype, THP-1 cells were treated with phorbol 12-myristate 13-acetate (PMA) at 100 ng/mL for 72 h.

### 2.3. Infection

Cells were infected with the described viruses at a MoI of 0.1 or 1.0 and the virus was adsorbed for 1 h. Mock infections were performed using a culture medium free of virus. At the designated time points, cell-free cell culture supernatants were harvested and used for total RNA extraction using the QIAamp viral RNA mini kit (Qiagen, Hilden, Germany) and for titration of infectious viruses via FFU assay. Adherent cells were washed in PBS and lysed in RIPA buffer (Thermo Fisher Scientific, Waltham, MA, USA) for immunoblotting or in RA1 buffer (Macherey-Nagel, Düren, Germany) for RNA-extraction using the NucleoSpin RNA Kit (Macherey-Nagel).

### 2.4. Focus Forming Unit Assay

Quantification of infectious viruses in cell culture supernatants was done by means of FFU assay, as described previously [35]. Briefly, Vero E6 cells were seeded in black optical-bottom 96-well plates. Confluent monolayers were incubated with decadal dilutions of samples. Viruses were allowed to adhere for 1 h, before the cells were overlaid with medium containing 3.2% carboxymethyl cellulose. At 7 d p.i. the cells were washed in PBS, fixed with ice-cold methanol, and foci of infected cells were detected using anti-hantavirus nucleocapsid antibody (ab34757, Abcam, Cambridge, UK) and Alexa Fluor^®^ 488 conjugated secondary antibody (Anti-mouse IgG (H + L), F(ab’)2 Fragment Alexa Fluor^®^ 488 Conjugate, Cell Signaling Technology, Danvers, MA, USA). Foci of infected cells were counted using a fluorescence microscope. All samples were tested in quadruplicate.

### 2.5. Quantitative Reverse Transcription PCR

The isolated RNA from cell culture supernatants was used as template in one-step quantitative reverse transcription (qRT) PCR analysis using the Ambion AgPath-ID Kit (Thermo Fisher Scientific). Viral nucleic acids in the cell culture supernatant were quantified using PUUV- or TULV-specific qPCR assays [37]. Isolated RNA from cultivated cells was converted to cDNA using SuperScript IV reverse transcriptase (Thermo Fisher Scientific) and random primer mix (New England Biolabs, Ipswich, MA, USA). Analysis of host cell gene expression was performed using commercially available TaqMan^®^ Gene Expression Assays (Thermo Fisher Scientific; *IFNB1* (Hs01077958_s1); *IFNA1* (Hs04189288_g1); *IFNAR1* (Hs01066116_m1); *IFNAR2* (Hs01022059_m1); *IFNE* (Hs00703565_s1); *IFNK* (Hs00737883_m1); *IFNW1* (Hs00958789_s1); *IFNL1* (Hs00601677_g1); *IFNL2* (Hs00820125_g1); *IFNG* (Hs00989291_m1). *MYC* gene expression was measured as a reference gene for ∆c_T_ normalization, as described previously [38]. Each PCR setup included no-template controls.

### 2.6. Immunoblotting

Expression of the hantavirus nucleocapsid protein and of cellular proteins was analyzed via immunoblotting of whole cell lysates of infected cells. The hantavirus nucleocapsid protein was detected using an anti-hantavirus nucleocapsid protein antibody (Abcam; ab34757). Cellular Phospho-STAT1 was detected using Phospho-Stat1 (Tyr701) (D4A7). Rabbit mAb, PKR (EIF2AK2) was detected using the PKR (D7F7). Rabbit mAb, MxA (MX1) was detected using the MX1 (D3W7I) Rabbit mAb and b-Actin, respectively, GAPDH were detected using β-Actin Antibody and GAPDH (D16H11) XP^®^ Rabbit mAb (all: Cell Signaling Technology).

## 3. Results

### 3.1. Puumala Virus Replicates More Efficiently Than Tula Virus in IFN-Competent Cell Types

**Figure 1:**
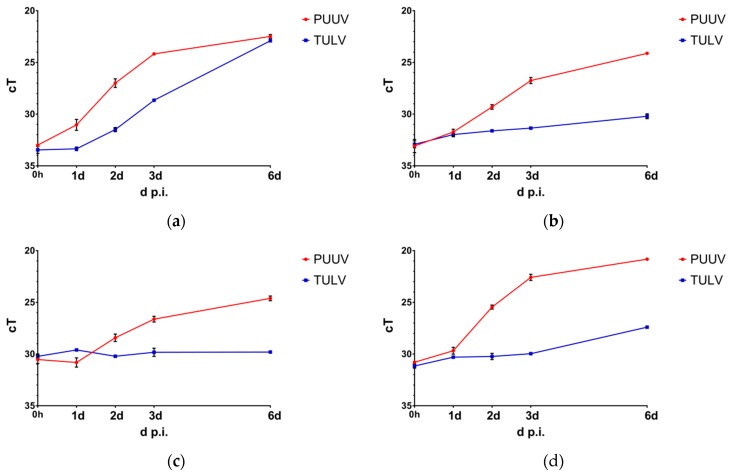
Quantification of viral RNA. The amplification of viral RNAs in supernatants of Puumala virus (PUUV)- and Tula virus (TULV)-infected (**a**) Vero E6, (**b**) A549, (**c**) HMEC-1, and (**d**) THP-1/PMA cells was quantified by means of qRT-PCR analysis of viral RNA copies in cell-free cell culture supernatants. Cells were infected with a multiplicity of infection (MoI) = 0.1 and viral RNA copies were quantified at 0 h, 1 d, 2 d, 3 d, and 6 d post infection.

In this study, we compared the replication of TULV and PUUV in human epithelial and endothelial cells, as well as in cells of the monocyte–macrophage lineage. The cells were infected with a multiplicity of infection (MoI) of 0.1 and viral RNA concentrations in the cell culture supernatants were quantified via qRT-PCR at 0 h to 6 d p.i. (Figure 1). All experiments were performed in duplicate using RNA samples from two independently infected cell cultures for each analysis.

As expected, both TULV and PUUV replicated successfully in the IFN-deficient Vero E6 cells. While PUUV replicated more rapidly in the beginning, at 6 d p.i. viral RNA quantities were comparable in the supernatants of TULV- and PUUV-infected Vero E6 cells. This was not the case in the lung epithelial A549 cells, where again a rapid amplification of PUUV RNA could be observed in the cell culture supernatants during the first 2 d p.i., which increased even more until 6 d post infection. In contrast, only a slight increase in TULV RNA concentrations could be observed until 6 d post infection. In the microvascular endothelial HMEC-1 cells, PUUV showed RNA amplification kinetics similar to those in the A549 cells, albeit the replication was overall less efficient. In TULV-infected HMEC-1 cells, no replication of viral RNAs could be observed during 6 d post infection. In the macrophage-like PMA-differentiated THP-1 cells, a rapid increase of PUUV RNA occurred in the supernatants during the first 3 d post infection. In contrast, viral RNA concentrations in the supernatants of TULV-infected THP-1/PMA cells increased only slightly between 3 d and 6 d post infection.

The RNA amplification kinetics in the selected endothelial and macrophage-like cell lines closely resembled those observed in primary cells (Figure S1). Similarly to the HMEC-1 and THP-1/PMA cells, amplification of PUUV RNA could also be observed in the supernatants of primary glomerular microvascular endothelial cells (HGMEC) and granulocyte-macrophage colony-stimulating factor (GM-CSF) polarized PBMC-derived M1 macrophages, whereas no replication of TULV was detectable in these cells. In contrast, in macrophage colony-stimulating factor (M-CSF) polarized PBMC-derived M2 macrophages, both viruses failed to replicate, as no increase of viral RNA in the cell culture supernatant was detectable during 6 d post infection.

**Figure 2:**
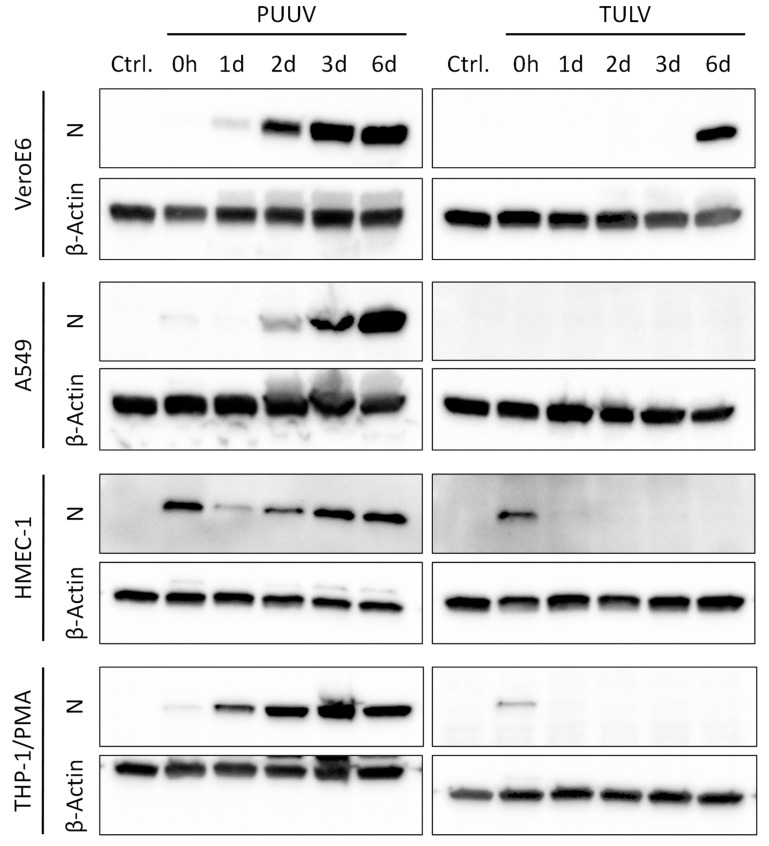
Viral nucleocaspid protein expression in PUUV- and TULV-infected cells. The expression of the viral nucleocapsid (N) protein was analyzed via immunoblotting in lysates of PUUV- or TULV-infected Vero E6, A549, HMEC-1, and THP-1/PMA cells. Cells were infected with a MoI = 0.1 and N expression was detected at 0 h, 1 d, 2 d, 3 d, and 6 d post infection. Detection of β-Actin served as loading control.

As an additional marker of viral replication, we analyzed the expression of the viral nucleocapsid (N) protein in cell lysates via immunoblotting (Figure 2). Again, a clear increase in N protein expression could be observed in the PUUV-infected cells, starting from 2 d p.i. in the Vero E6, A549, and HMEC-1 cells, or even 1 d p.i. in the THP-1/PMA cells. In contrast, a strong expression of the TULV N protein was only detectable in the Vero E6 cells at 6 d post infection.

The N protein expression in the cell lines was very similar to primary cells, proving the suitability of the selected cell lines as in vitro model systems of endothelial cells and macrophages. In the PUUV-infected M1 and M2 polarized macrophages, viral N protein expression increased rapidly during the first 2 d p.i., followed by a decrease between 3 d and 6 d p.i. (Figure S2). In the TULV-infected M1 and M2 polarized macrophages, no N expression was observable after the initial infection (0 h p.i.). In the HGMECs, only PUUV infection led to an accumulation of N protein from 2 d to 6 d post infection (Figure S3).

**Figure 3:**
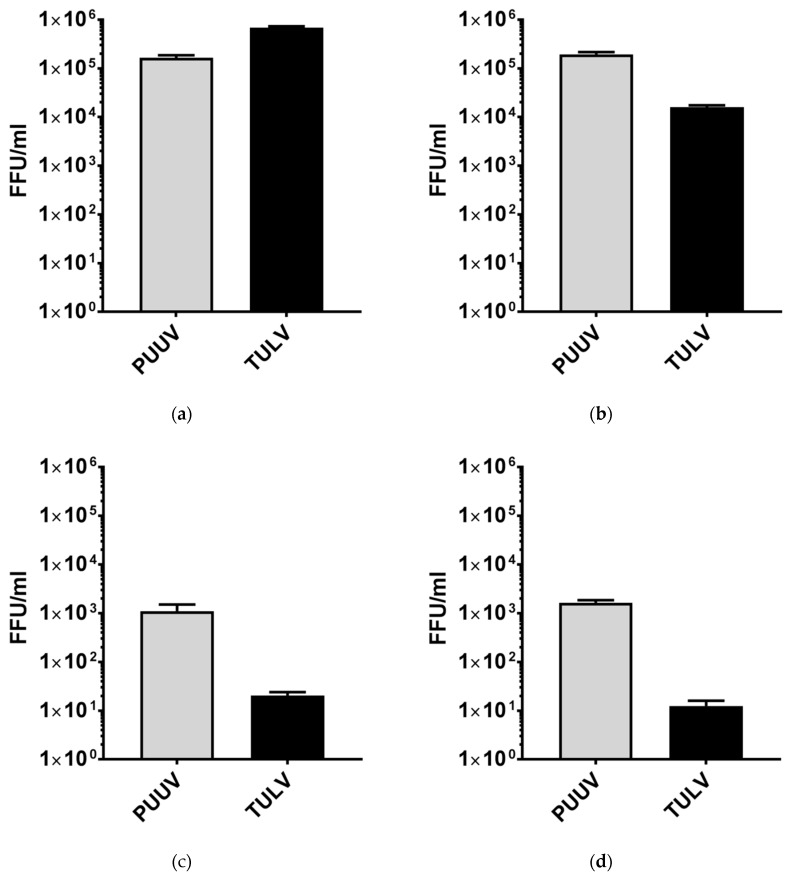
Progeny virus titers at 6 d p.i. with PUUV or TULV. The release of infectious viruses from PUUV- or TULV-infected (**a**) Vero E6, (**b**) A549, (**c**) HMEC-1, and (**d**) THP-1/PMA cells were quantified by focus forming unit (FFU) assay. Cells were infected with a MoI = 0.1 and infectious viruses were quantified in cell-free cell culture supernatants at 6 d post infection.

To verify that the increasing amounts of viral RNA in the supernatants and of viral N protein in the lysates of infected cells correlate with successful viral replication and the production and release of viral progeny, we quantified the concentrations of infectious virus particles in cell culture supernatants at 6 d p.i. via FFU assay (Figure 3). Again, we could prove that both viruses replicate efficiently in the Vero E6 cells, in which TULV titers even excelled those of PUUV. In the A549 cells, PUUV replicated to comparable titers as in the Vero E6 cells. However, TULV replicated less efficiently, reaching titers 10-fold lower than PUUV at 6 d p.i. and more than 40-fold lower when compared to the TULV titers in the Vero E6 cells. In the supernatants of THP-1/PMA and HMEC-1 cells, almost no infectious TULV particles were detectable at 6 d p.i., whereas PUUV replicated successfully, albeit PUUV titers were approximately 100-fold lower compared to the A549 and Vero E6 cells.

In combination, our results show clear differences in the kinetics of viral replication between pathogenic PUUV and the non- or low-pathogenic TULV. Both viruses efficiently infected IFN-deficient Vero E6 cells and replicated to comparable titers. However, a faster onset of viral RNA amplification and a more pronounced accumulation of viral N protein were observed in the PUUV-infected cells. This became even more evident in the A549 lung epithelial cells, where TULV replication was less vigorous when compared to PUUV, leading to significantly reduced titers at 6 d post infection. The endothelial HMEC-1 and the macrophage-like THP-1/PMA cells supported PUUV replication less well than A549 or Vero E6 cells. Still, a rapid onset of viral N protein expression and efficient production and release of viral progeny could be detected, while TULV replication was very weak or even undetectable in these cells.

### 3.2. PUUV Infection Induces IFN-β and IFN-λ Gene Expression

To further investigate the reasons underlying the inefficient TULV replication in all cells except IFN-deficient Vero E6 cells, we analyzed the IFN response towards PUUV and TULV infection. To do so, the expression of cellular IFN genes was quantified via qRT-PCR in TULV-, PUUV- or mock-infected HMEC-1, and THP-1/PMA cells (MoI = 1.0) at 0 h to 48 h post infection.

Expression of IFN-α1 (*IFNA1*) was not detectable in the THP-1/PMA cells and only weakly in the HMEC-1 cells, in which *IFNA1* gene expression was comparable in infected vs. mock-infected cells (data not shown). Likewise, expression of IFN-ε (*IFNE*) was weakly detectable in HMEC-1 cells but showed a comparable progression in infected and mock-infected cells. Expression of IFN-ω (*IFNW1*), IFN-κ (*IFNK*) and IFN-γ (*IFNG*) was not detectable at all (data not shown). Expression of the type I IFN receptors *IFNAR1* and *IFNAR2* was detected in the HMEC-1 cells but expression was not altered by PUUV or TULV infection (data not shown).

**Figure 4:**
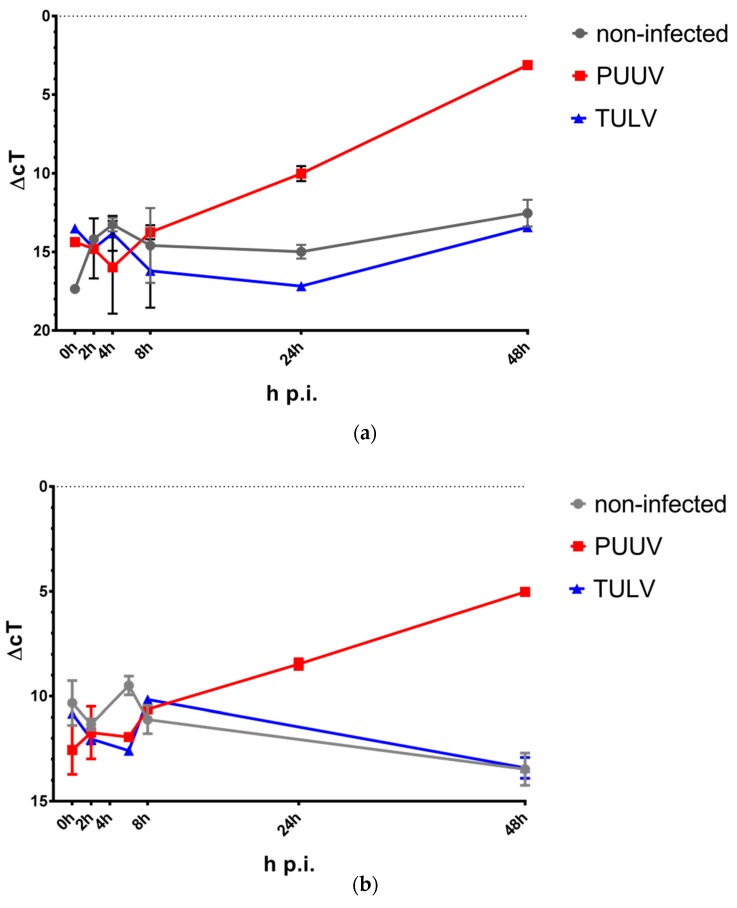
*IFNB1* gene expression in response to infection. Expression of the *IFNB1* gene was quantified in response to PUUV or TULV infection of (**a**) HMEC-1 or (**b**) THP-1 cells via qRT-PCR and normalized to the expression of the cellular *MYC* gene. Cells were infected using a MoI of 1.0 and lysed at the indicated time points. No *IFNB1* expression was detectable at 24 h p.i. in non-infected or TULV-infected THP-1/PMA cells.

However, a strong induction of IFN-β (*IFNB1*) expression could be observed at 24 h and 48 h following PUUV infection of HMEC-1 and THP-1/PMA cells (Figure 4). Similarly, IFN-λ (*IFNL1* and *IFNL2*) expression increased at 48 h post PUUV infection, being mostly non-detectable in TULV- or mock-infected samples or at earlier time points (Figure S4).

### 3.3. PUUV but Not TULV Infection Induces Phosphorylation of STAT1 and Interferon-stimulated Gene Expression in Endothelial Cells and Monocytes/Macrophages

**Figure 5:**
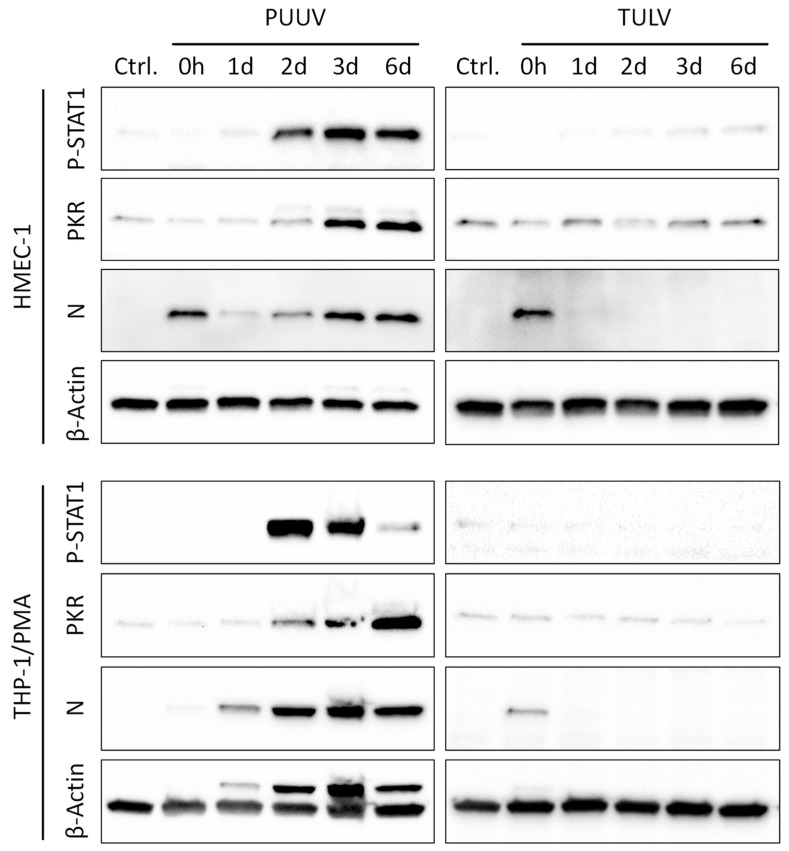
Interferon-stimulated protein expression in PUUV- and TULV-infected cells. The phosphorylation of STAT1 (Tyr701) and the expression of the cellular antiviral dsRNA-activated protein kinase PKR (EIF2AK2) was analyzed in lysates of PUUV- or TULV-infected HMEC-1 and THP-1/PMA cells. Expression of the viral nucleocapsid (N) protein was detected to monitor infection and cellular β-Actin served as loading control. Cells were infected with a MoI of 0.1 and protein expression was detected at 0 h, 1 d, 2 d, 3 d, and 6 d p.i. or in non-infected cells.

Subsequently, we analyzed the phosphorylation and activation of the IFN-responsive transcription factor STAT1 and the expression of the antiviral IFN-induced dsRNA-activated protein kinase (PKR) in lysates of PUUV- or TULV-infected HMEC-1 and THP-1/PMA cells via immunoblotting (Figure 5). In congruence with the stimulation of IFN gene expression, STAT1 phosphorylation was evident starting from 48 h p.i. in the PUUV-infected cells. This correlated with an accumulation of the viral N protein in PUUV-infected cells and also with a subsequent induction of IFN-induced PKR expression at 3 d to 6 d post infection. No increase in STAT1 phosphorylation or PKR expression could be observed until 6 d following TULV infection of HMEC-1 or THP-1/PMA cells and no viral N protein was detectable in TULV-infected cells after the initial infection (0 h p.i.).

These data indicate that TULV replication is most likely not hampered by an early induction of an IFN response in the HMEC-1 and THP-1/PMA cells. Instead, our data shows that the more vigorous PUUV infection of these cells induces a much stronger IFN response than the weak or abortive TULV infection. This is also supported by the detection of a strong MxA (MX1) expression in PUUV- but not in TULV-infected HGMECs (Figure S3).

### 3.4. TULV Replication Induces Only a Weak IFN Response in Permissive A549 Cells in Comparison to PUUV

**Figure 6:**
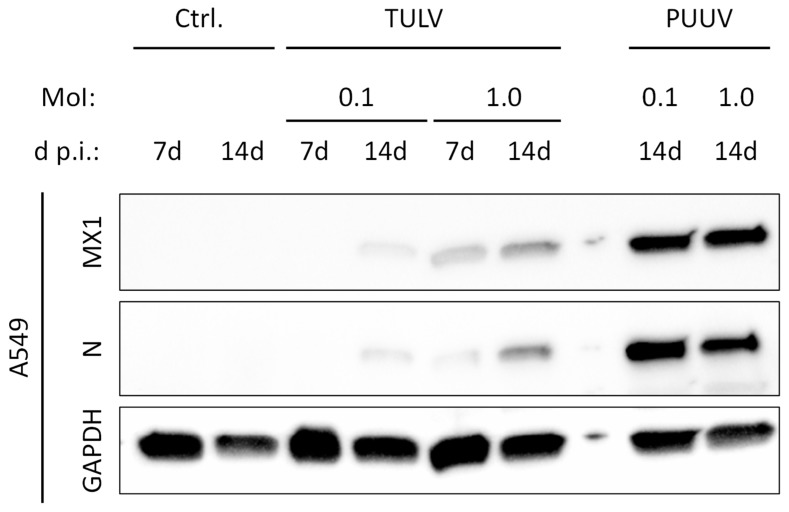
IFN-stimulated MX1 expression in PUUV- and TULV-infected cells. The expression of the cellular IFN-stimulated protein MX1 was analyzed in lysates of PUUV- or TULV-infected A549 cells. Expression of the viral nucleocapsid (N) protein was detected to monitor infection and cellular GAPDH served as loading control. Cells were infected with a MoI of 0.1 or 1.0 and protein expression was detected at 7 d or 14 d post infection.

TULV did not induce a detectable IFN response in the HMEC-1 and THP-1/PMA cells. However, productive replication of TULV was also not detectable in these cells. Therefore, we aimed to analyze if successful TULV replication in A549 cells may induce an IFN response comparable to that triggered by the actively replicating PUUV. To compensate for the slower replication of TULV in the A549 cells, we analyzed the expression of the IFN-stimulated antiviral MxA (MX1) protein following TULV or PUUV infection with a MoI of either 0.1 or 1.0 at 7 d and 14 d p.i. (Figure 6). In the cells infected with TULV at a MoI of 0.1, even after 14 d of infection, almost no N protein expression was detectable. In contrast to mock-infected cells, MX1 expression was weakly detectable at 14 d post TULV infection. MX1 and N expression were more pronounced following TULV infection at a MoI of 1.0, however both were still considerably weaker than in the PUUV-infected cells.

We conclude that active replication of TULV induces an IFN response in infected cells. However, in comparison to PUUV, TULV replication is much less efficient in most of the tested cell types, as can be seen by the weaker expression of viral N protein and viral RNAs. Most likely, this reduced accumulation of virus-derived immunostimulatory molecular patterns results in the weaker IFN response towards TULV in contrast to PUUV infection.

## 4. Discussion

While many hantaviruses are pathogenic to humans, some—like PHV—are considered non-pathogenic [20]. PUUV is the most important cause of HFRS in Europe, causing around 10,000 infections per year [2]. In contrast, the pathogenicity of the widely distributed TULV is still unclear. Although serological data shows that human infections occur, only a few reports of disease caused by TULV exist [8,9,10,11,12]. The mechanisms underlying the various degrees of hantavirus pathogenicity are still unknown. However, solid evidence exists that pathogenic and non-pathogenic hantaviruses differ in their interaction with the host immune system and both innate and adaptive immune responses have been shown to contribute to the hantavirus-associated disease [17,18,20,39].

The initial sensing of a hantavirus infection by the innate immune system is mediated through germ-line encoded pattern recognition receptors (PRRs). Recognition of hantaviral RNA species via endosomal Toll-like receptor-3 (TLR-3) [40] and the cytoplasmic retinoic acid-inducible gene I (RIG-I) RNA helicase [30] induces the activation of signaling cascades that lead to the production and secretion of IFNs, which in turn induce the expression of IFN-stimulated genes (ISGs) like antiviral MxA (MX1) [18]. Three types of IFN have been described to date (I, II, and III), which are categorized by the type of receptors they use for signaling. The type I IFN family includes IFN-α, IFN-β, IFN-ɛ, IFN-κ, IFN-τ, and IFN-ω, and signal via the IFNAR1 and IFNAR2 receptor chains. IFN-γ is the sole member of the type II family, signaling through IFNGR1 and IFNGR2. Type III IFNs are the latest addition to the IFN family. The four members (IFN-λ1–IFN-λ4) signal via a receptor complex involving IL-10R2 and IL-28Ra subunits (reviewed in [41]). Type I and type III IFNs are expressed following detection of pathogens by PRRs, and the signaling pathways that induce type I and type III IFNs largely overlap, although a differential requirement for IRFs and NF-κB in the induction of type I and type III IFNs has been described (reviewed in [42]). The expression of different IFN types is tissue dependent and, in our hands, only IFN-β and IFN-λ were induced by hantavirus infection in the studied cell types. Early activation of the IFN response during hantavirus infection is sufficient to block viral replication. Therefore, for hantaviruses to be pathogenic, they need to prevent or at least delay early IFN induction [17,18,19]. A delayed induction of an excessive innate immune response however is unable to control the infection and is believed to contribute to pathogenesis [26,43,44,45].

In this study, we investigated and compared the replication of the pathogenic PUUV and presumably non- or low-pathogenic TULV in vitro in different cell types and analyzed the induction of innate immune responses by both viruses. PUUV has been previously reported to have a wide in vitro host cell range, replicating successfully not only in IFN-deficient Vero cells, but also in human umbilical vein endothelial cells (HUVECs) [46,47], in primary human monocytes/macrophages and PMA-differentiated THP-1 cells [48], as well as in primary human kidney cells [49] and several established cell lines [50]. However, PUUV replication in human cells is usually less efficient than in cells of its natural host or IFN-deficient Vero E6 cells [50].

TULV has been reported to replicate efficiently in IFN-deficient Vero E6 cells [8,51], also it has been shown to induce apoptosis in these cells [52]. Furthermore, like PHV, and in contrast to the Hantaan virus (HTNV), TULV has been previously shown to be unable to replicate efficiently in the human megakaryocytic HEL cell line [53]. There are conflicting results concerning the capability of TULV to replicate in HUVECs. One study shows inefficient TULV replication in HUVECs in comparison to HTNV, which was accompanied by weaker IFN-β induction, most likely due to the less efficient replication [54]. In contrast, results from another group show successful replication of TULV in HUVECs, which was accompanied by a strong induction of the ISGs MxA (MX1) and ISG56 (IFIT1) [55]. In our hands, TULV was not able to replicate efficiently in endothelial cells. This was independent of the TULV strain, as similar results were observed for the TULV strains Moravia-5302Ma-94 and Lodz (Figure S5). Similarly, no efficient replication of TULV was observed in the macrophage-like PMA-treated THP-1 cells, while only weak replication of TULV was observed in A549 cells. Inefficient replication of TULV in A549 cells has also been reported by Shim et al. [33], who also show that non-pathogenic hantaviruses are less resistant to the antiviral activities induced by IFN-β.

The inefficient replication of TULV is in stark contrast to PUUV, which replicated productively in all tested cell types, albeit replication was strongest in the IFN-deficient Vero E6 cells. In all tested cell types, PUUV infection resulted in a strong IFN response at approximately 48 h p.i., which has been described by previous reports as well [30,43,56] (also reviewed in [18,19,20]). Receptor binding of type I or III IFNs results in phosphorylation of STAT1, which induces STAT1 dimerization, nuclear translocation, and IFN-stimulated response elements (ISRE)-dependent gene expression of ISGs [57]. Consistent with this, we could show that induction of IFN-β and IFN-λ expression in PUUV-infected cells was accompanied by subsequent phosphorylation of STAT1 and increased expression of PKR and MX1. This IFN response towards PUUV infection was dependent on active viral replication and was not caused by residual IFNs in viral stock preparations, which was verified by infection experiments using UV-irradiated PUUV stocks or stocks depleted of infectious viruses via exclusion filtration (300 kDa) [58]. Neither infection with PUUV stocks inactivated via UV-irradiation nor treatment with PUUV stocks depleted of infectious viral particles resulted in a detectable IFN response (Figure S6).

The inability of a non-pathogenic hantavirus to replicate in human endothelial cells has been described before in the case of PHV. The non-pathogenic PHV has been shown to be unable to replicate in HUVECs, which is caused by an early IFN induction triggered by PHV infection [21]. In contrast to this, we did not observe a strong early IFN response towards TULV infection of HMEC-1 and THP-1/PMA cells. This is in agreement with previous reports showing that TULV—in contrast to PHV—is able to inhibit early IFN induction through its Gn protein cytoplasmic tail (Gn-T), which blocks RIG-I- and TBK1-directed transcription from ISREs and IFN-β promoters [55]. Furthermore, TULV, like PUUV, encodes a functional non-structural NSs protein, which is believed to play a role in IFN regulation [59,60]. In contrast to PUUV, TULV infection did also not induce a substantial IFN response between 2 d and 6 d post infection. This non-detectable IFN induction may be explained by the weak or even undetectable replication of TULV in comparison to PUUV. This is also supported by the fact that both PUUV and TULV induce IFN-λ expression in Vero E6 cells (Figure S7), in which both viruses replicate to similar titers. While being IFN-α/β incompetent [61], previous reports have already described IFN-λ production by hantavirus-infected Vero E6 cells [34,62]. Furthermore, our results indicate that the slower and less-efficient replication of TULV in A549 cells is also accompanied by a much less severe induction of innate immune responses by TULV in comparison to PUUV in these cells, supporting the notion that the more vigorous PUUV replication triggers the stronger IFN response in these cells. These results are also in agreement with our previous data, showing that TULV infection of A549 cells is less IFN-stimulatory than infection with pathogenic DOBV [35].

Except for differences in the viruses ability to modulate the innate immune response towards infection, the more efficient replication of PUUV compared to TULV may also be explained by different entry mechanisms, as PUUV uses α_V_β_3_ and TULV α_5_β_1_ integrins for entry (reviewed in [20]). However, we found the expression of β_1_ integrins to be comparable in THP-1/PMA, A549, and HMEC-1 cells—and even slightly stronger than in Vero E6 cells—and integrin α_5_ to be strongly expressed in the THP-1/PMA and HMEC-1 cells (Figure S8). Therefore, a lack of receptor expression is most likely not the cause of the inefficient TULV replication in THP-1/PMA and HMEC-1 cells.

Of course it has to be considered that phenotypic differences observed in vitro may at least in part be caused by accumulation of natural mutations, acquired during propagation in cell culture, as it has been shown for different substrains of PUUV [63]. To exclude this possibility, we also analyzed the replication of the TULV strain Lodz and PUUV strain CG1820 in HMEC-1 cells. While PUUV CG1820 replicated in HMEC-1 cells, TULV Lodz—like TULV Moravia-5302Ma-94—failed to replicate efficiently within 6 d post infection. Furthermore, while PUUV CG1820 strongly induced an IFN response upon infection of A549 cells, this was not the case following infection with TULV Lodz, as with TULV Moravia-5302Ma-94 (Figures S5 and S9).

In conclusion, we could show major differences between the pathogenic PUUV and the presumably non- or low-pathogenic TULV regarding their in vitro replication efficiency in different cell types. PUUV replicates successfully in all tested cell types except for M2-like polarized macrophages. In contrast, TULV showed efficient replication only in IFN-deficient Vero E6 cells, while TULV replication in IFN-competent A549 lung epithelial cells was considerably weaker and no viral replication could be detected in the endothelial HMEC-1 or in the macrophage-like cells. Productive PUUV replication was characterized by an accumulation of viral N protein in infected cells. Strong expression of the viral N protein was detectable approximately 48 h p.i., which correlated with the onset of a strong IFN response towards PUUV infection. Following TULV infection, a comparably weaker expression of the N protein was observed, even in the permissive Vero E6 and A549 cells. This correlated with a slower viral replication and also a more delayed and weaker IFN response towards TULV infection. Our results underline the complexity of the interplay between virus and innate immune responses. As the virulence of viruses is often determined by replication kinetics, the less efficient replication of TULV in endothelial cells and macrophages may be one of the factors explaining the presumably lower pathogenicity of TULV compared to PUUV, at least if these in vitro observations are reflective of the more complex in vivo situation. However, the differences in virus host cell interactions that determine the less efficient TULV replication need further investigation, as this could not be explained by an early IFN response towards TULV infection or a lack of expression of cellular α_5_β_1_ integrins required for TULV entry. Moreover, further attempts have to be done to understand if the strong IFN response caused by PUUV in vitro may possibly contribute to immunopathology in vivo or rather distinguishes the moderately pathogenic PUUV from other highly pathogenic hantaviruses.

## Supplementary Materials

The following are available online at https://www.mdpi.com/1999-4915/11/9/855/s1, Figure S1: Quantification of viral RNA amplification in PUUV- and TULV-infected peripheral blood mononuclear cell (PBMC)-derived macrophages and human glomerular microvascular endothelial cells (HGMEC), Figure S2: Nucleocaspid protein expression in PUUV- and TULV-infected PBMC-derived M1 and M2 polarized macrophages, Figure S3: Nucleocaspid protein and MX1 expression in PUUV- and TULV-infected HGMECs, Figure S4: IFN-λ gene expression in response to PUUV or TULV infection, Figure S5: RNA replication kinetics of different PUUV and TULV strains, Figure S6: Nucleocaspid protein and MX1 expression in A549 cells infected with PUUV, UV-irradiated PUUV, or PUUV stocks depleted of infectious viruses, Figure S7: IFNL1 gene expression in PUUV- or TULV-infected Vero E6 cells, Figure S8: Integrin expression in THP-1/PMA, A549, HMEC-1, and Vero E6 cells, Figure S9: IRF-activity in PUUV- and TULV-infected A549-Dual™ reporter cells.

## References

[1] Kruger D.H., Figueiredo L.T., Song J.W., Klempa B. (2015). Hantaviruses—Globally emerging pathogens. J. Clin. Virol..

[2] Vaheri A., Henttonen H., Voutilainen L., Mustonen J., Sironen T., Vapalahti O. (2013). Hantavirus infections in Europe and their impact on public health. Rev. Med. Virol..

[3] Makary P., Kanerva M., Ollgren J., Virtanen M.J., Vapalahti O., Lyytikainen O. (2010). Disease burden of Puumala virus infections, 1995–2008. Epidemiol. Infect..

[4] Kruger D.H., Ulrich R.G., Hofmann J. (2013). Hantaviruses as zoonotic pathogens in Germany. Dtsch. Arztebl. Int..

[5] Maas M., de Vries A., van Roon A., Takumi K., van der Giessen J., Rockx B. (2017). High Prevalence of Tula Hantavirus in Common Voles in The Netherlands. Vector Borne Zoonotic Dis..

[6] Vapalahti O., Mustonen J., Lundkvist A., Henttonen H., Plyusnin A., Vaheri A. (2003). Hantavirus infections in Europe. Lancet Infect. Dis..

[7] Schmidt-Chanasit J., Essbauer S., Petraityte R., Yoshimatsu K., Tackmann K., Conraths F.J., Sasnauskas K., Arikawa J., Thomas A., Pfeffer M. (2010). Extensive host sharing of central European Tula virus. J. Virol..

[8] Vapalahti O., Lundkvist A., Kukkonen S.K., Cheng Y., Gilljam M., Kanerva M., Manni T., Pejcoch M., Niemimaa J., Kaikusalo A. (1996). Isolation and characterization of Tula virus, a distinct serotype in the genus Hantavirus, family Bunyaviridae. J. Gen. Virol..

[9] Mertens M., Hofmann J., Petraityte-Burneikiene R., Ziller M., Sasnauskas K., Friedrich R., Niederstrasser O., Kruger D.H., Groschup M.H., Petri E. (2011). Seroprevalence study in forestry workers of a non-endemic region in eastern Germany reveals infections by Tula and Dobrava-Belgrade hantaviruses. Med. Microbiol. Immunol..

[10] Klempa B., Meisel H., Rath S., Bartel J., Ulrich R., Kruger D.H. (2003). Occurrence of renal and pulmonary syndrome in a region of northeast Germany where Tula hantavirus circulates. J. Clin. Microbiol..

[11] Zelena H., Mrazek J., Kuhn T. (2013). Tula hantavirus infection in immunocompromised host, Czech Republic. Emerg. Infect. Dis..

[12] Reynes J.M., Carli D., Boukezia N., Debruyne M., Herti S. (2015). Tula hantavirus infection in a hospitalised patient, France, June 2015. Euro Surveill..

[13] Clement J., Van Ranst M. (2016). Three vole species and one (?) novel arvicolid hantavirus pathogen: Tula virus revisited. Euro Surveill..

[14] Hepojoki J., Vaheri A., Strandin T. (2014). The fundamental role of endothelial cells in hantavirus pathogenesis. Front. Microbiol..

[15] Mackow E.R., Gavrilovskaya I.N. (2009). Hantavirus regulation of endothelial cell functions. Thromb. Haemost..

[16] Krautkramer E., Zeier M. (2014). Old World hantaviruses: Aspects of pathogenesis and clinical course of acute renal failure. Virus Res..

[17] Mackow E.R., Dalrymple N.A., Cimica V., Matthys V., Gorbunova E., Gavrilovskaya I. (2014). Hantavirus interferon regulation and virulence determinants. Virus Res..

[18] Matthys V., Mackow E.R. (2012). Hantavirus regulation of type I interferon responses. Adv. Virol..

[19] Rang A. (2010). Modulation of innate immune responses by hantaviruses. Crit. Rev. Immunol..

[20] Ermonval M., Baychelier F., Tordo N. (2016). What Do We Know about How Hantaviruses Interact with Their Different Hosts?. Viruses.

[21] Alff P.J., Gavrilovskaya I.N., Gorbunova E., Endriss K., Chong Y., Geimonen E., Sen N., Reich N.C., Mackow E.R. (2006). The pathogenic NY-1 hantavirus G1 cytoplasmic tail inhibits RIG-I- and TBK-1-directed interferon responses. J. Virol..

[22] Geimonen E., Neff S., Raymond T., Kocer S.S., Gavrilovskaya I.N., Mackow E.R. (2002). Pathogenic and nonpathogenic hantaviruses differentially regulate endothelial cell responses. Proc. Natl. Acad. Sci. USA.

[23] Brocato R.L., Wahl V., Hammerbeck C.D., Josleyn M.D., McElroy A.K., Smith J.M., Hooper J.W. (2018). Innate immune responses elicited by Sin Nombre virus or type I IFN agonists protect hamsters from lethal Andes virus infections. J. Gen. Virol..

[24] Safronetz D., Zivcec M., Lacasse R., Feldmann F., Rosenke R., Long D., Haddock E., Brining D., Gardner D., Feldmann H. (2011). Pathogenesis and host response in Syrian hamsters following intranasal infection with Andes virus. PLoS Pathog..

[25] Schonrich G., Kruger D.H., Raftery M.J. (2015). Hantavirus-induced disruption of the endothelial barrier: Neutrophils are on the payroll. Front. Microbiol..

[26] Sundstrom K.B., Nguyen Hoang A.T., Gupta S., Ahlm C., Svensson M., Klingstrom J. (2016). Andes Hantavirus-Infection of a 3D Human Lung Tissue Model Reveals a Late Peak in Progeny Virus Production Followed by Increased Levels of Proinflammatory Cytokines and VEGF-A. PLoS ONE.

[27] Ades E.W., Candal F.J., Swerlick R.A., George V.G., Summers S., Bosse D.C., Lawley T.J. (1992). HMEC-1: Establishment of an immortalized human microvascular endothelial cell line. J. Investig. Dermatol..

[28] Munoz-Vega M., Masso F., Paez A., Carreon-Torres E., Cabrera-Fuentes H.A., Fragoso J.M., Perez-Hernandez N., Martinez L.O., Najib S., Vargas-Alarcon G. (2018). Characterization of immortalized human dermal microvascular endothelial cells (HMEC-1) for the study of HDL functionality. Lipids Health Dis..

[29] Xu Y., Swerlick R.A., Sepp N., Bosse D., Ades E.W., Lawley T.J. (1994). Characterization of expression and modulation of cell adhesion molecules on an immortalized human dermal microvascular endothelial cell line (HMEC-1). J. Investig. Dermatol..

[30] Lee M.H., Lalwani P., Raftery M.J., Matthaei M., Lutteke N., Kirsanovs S., Binder M., Ulrich R.G., Giese T., Wolff T. (2011). RNA helicase retinoic acid-inducible gene I as a sensor of Hantaan virus replication. J. Gen. Virol..

[31] Oelschlegel R., Kruger D.H., Rang A. (2007). MxA-independent inhibition of Hantaan virus replication induced by type I and type II interferon in vitro. Virus Res..

[32] Popugaeva E., Witkowski P.T., Schlegel M., Ulrich R.G., Auste B., Rang A., Kruger D.H., Klempa B. (2012). Dobrava-Belgrade hantavirus from Germany shows receptor usage and innate immunity induction consistent with the pathogenicity of the virus in humans. PLoS ONE.

[33] Shim S.H., Park M.S., Moon S., Park K.S., Song J.W., Song K.J., Baek L.J. (2011). Comparison of innate immune responses to pathogenic and putative non-pathogenic hantaviruses in vitro. Virus Res..

[34] Stoltz M., Klingstrom J. (2010). Alpha/beta interferon (IFN-alpha/beta)-independent induction of IFN-lambda1 (interleukin-29) in response to Hantaan virus infection. J. Virol..

[35] Witkowski P.T., Bourquain D., Bankov K., Auste B., Dabrowski P.W., Nitsche A., Kruger D.H., Schaade L. (2016). Infection of human airway epithelial cells by different subtypes of Dobrava-Belgrade virus reveals gene expression patterns corresponding to their virulence potential. Virology.

[36] Gavrilovskaya I.N., Chumakov M.P., Apekina N.S., Ryltseva E.V., Martiyanova L.I., Gorbachkova E.A., Bernshtein A.D., Zakharova M.A., Boiko V.A. (1983). Adaptation to laboratory and wild animals of the haemorrhagic fever with renal syndrome virus present in the foci of European U.S.S.R. Brief report. Arch. Virol..

[37] Kramski M., Meisel H., Klempa B., Kruger D.H., Pauli G., Nitsche A. (2007). Detection and typing of human pathogenic hantaviruses by real-time reverse transcription-PCR and pyrosequencing. Clin. Chem..

[38] Kramski M., Matz-Rensing K., Stahl-Hennig C., Kaup F.J., Nitsche A., Pauli G., Ellerbrok H. (2010). A novel highly reproducible and lethal nonhuman primate model for orthopox virus infection. PLoS ONE.

[39] Schonrich G., Rang A., Lutteke N., Raftery M.J., Charbonnel N., Ulrich R.G. (2008). Hantavirus-induced immunity in rodent reservoirs and humans. Immunol. Rev..

[40] Handke W., Oelschlegel R., Franke R., Kruger D.H., Rang A. (2009). Hantaan virus triggers TLR3-dependent innate immune responses. J. Immunol..

[41] Xi Y., Day S.L., Jackson R.J., Ranasinghe C. (2012). Role of novel type I interferon epsilon in viral infection and mucosal immunity. Mucosal Immunol..

[42] Hemann E.A., Gale M., Savan R. (2017). Interferon Lambda Genetics and Biology in Regulation of Viral Control. Front. Immunol..

[43] Strandin T., Hepojoki J., Laine O., Makela S., Klingstrom J., Lundkvist A., Julkunen I., Mustonen J., Vaheri A. (2016). Interferons Induce STAT1-Dependent Expression of Tissue Plasminogen Activator, a Pathogenicity Factor in Puumala Hantavirus Disease. J. Infect. Dis..

[44] Raftery M.J., Lalwani P., Krautkrmer E., Peters T., Scharffetter-Kochanek K., Kruger R., Hofmann J., Seeger K., Kruger D.H., Schonrich G. (2014). beta2 integrin mediates hantavirus-induced release of neutrophil extracellular traps. J. Exp. Med..

[45] Strandin T., Makela S., Mustonen J., Vaheri A. (2018). Neutrophil Activation in Acute Hemorrhagic Fever With Renal Syndrome Is Mediated by Hantavirus-Infected Microvascular Endothelial Cells. Front. Immunol..

[46] Yanagihara R., Silverman D.J. (1990). Experimental infection of human vascular endothelial cells by pathogenic and nonpathogenic hantaviruses. Arch. Virol..

[47] Goeijenbier M., Meijers J.C., Anfasa F., Roose J.M., van de Weg C.A., Bakhtiari K., Henttonen H., Vaheri A., Osterhaus A.D., van Gorp E.C. (2015). Effect of Puumala hantavirus infection on human umbilical vein endothelial cell hemostatic function: Platelet interactions, increased tissue factor expression and fibrinolysis regulator release. Front. Microbiol..

[48] Temonen M., Lankinen H., Vapalahti O., Ronni T., Julkunen I., Vaheri A. (1995). Effect of interferon-alpha and cell differentiation on Puumala virus infection in human monocyte/macrophages. Virology.

[49] Krautkramer E., Grouls S., Stein N., Reiser J., Zeier M. (2011). Pathogenic old world hantaviruses infect renal glomerular and tubular cells and induce disassembling of cell-to-cell contacts. J. Virol..

[50] Temonen M., Vapalahti O., Holthofer H., Brummer-Korvenkontio M., Vaheri A., Lankinen H. (1993). Susceptibility of human cells to Puumala virus infection. J. Gen. Virol..

[51] Kanerva M., Melen K., Vaheri A., Julkunen I. (1996). Inhibition of puumala and tula hantaviruses in Vero cells by MxA protein. Virology.

[52] Li X.D., Kukkonen S., Vapalahti O., Plyusnin A., Lankinen H., Vaheri A. (2004). Tula hantavirus infection of Vero E6 cells induces apoptosis involving caspase 8 activation. J. Gen. Virol..

[53] Lutteke N., Raftery M.J., Lalwani P., Lee M.H., Giese T., Voigt S., Bannert N., Schulze H., Kruger D.H., Schonrich G. (2010). Switch to high-level virus replication and HLA class I upregulation in differentiating megakaryocytic cells after infection with pathogenic hantavirus. Virology.

[54] Kraus A.A., Raftery M.J., Giese T., Ulrich R., Zawatzky R., Hippenstiel S., Suttorp N., Kruger D.H., Schonrich G. (2004). Differential antiviral response of endothelial cells after infection with pathogenic and nonpathogenic hantaviruses. J. Virol..

[55] Matthys V., Gorbunova E.E., Gavrilovskaya I.N., Pepini T., Mackow E.R. (2011). The C-terminal 42 residues of the Tula virus Gn protein regulate interferon induction. J. Virol..

[56] Resman Rus K., Korva M., Bogovic P., Pal E., Strle F., Avsic-Zupanc T. (2018). Delayed Interferon Type 1-Induced Antiviral State Is a Potential Factor for Hemorrhagic Fever With Renal Syndrome Severity. J. Infect. Dis..

[57] Schneider W.M., Chevillotte M.D., Rice C.M. (2014). Interferon-stimulated genes: A complex web of host defenses. Annu. Rev. Immunol..

[58] Kraus A.A., Priemer C., Heider H., Kruger D.H., Ulrich R. (2005). Inactivation of Hantaan virus-containing samples for subsequent investigations outside biosafety level 3 facilities. Intervirology.

[59] Jaaskelainen K.M., Kaukinen P., Minskaya E.S., Plyusnina A., Vapalahti O., Elliott R.M., Weber F., Vaheri A., Plyusnin A. (2007). Tula and Puumala hantavirus NSs ORFs are functional and the products inhibit activation of the interferon-beta promoter. J. Med. Virol..

[60] Jaaskelainen K.M., Plyusnina A., Lundkvist A., Vaheri A., Plyusnin A. (2008). Tula hantavirus isolate with the full-length ORF for nonstructural protein NSs survives for more consequent passages in interferon-competent cells than the isolate having truncated NSs ORF. Virol. J..

[61] Osada N., Kohara A., Yamaji T., Hirayama N., Kasai F., Sekizuka T., Kuroda M., Hanada K. (2014). The genome landscape of the african green monkey kidney-derived vero cell line. DNA Res..

[62] Prescott J., Hall P., Acuna-Retamar M., Ye C., Wathelet M.G., Ebihara H., Feldmann H., Hjelle B. (2010). New World hantaviruses activate IFNlambda production in type I IFN-deficient vero E6 cells. PLoS ONE.

[63] Sundstrom K.B., Stoltz M., Lagerqvist N., Lundkvist A., Nemirov K., Klingstrom J. (2011). Characterization of two substrains of Puumala virus that show phenotypes that are different from each other and from the original strain. J. Virol..

