# Supplementary material for: Puumala and Tula Virus Differ in Replication Kinetics and Innate Immune Stimulation in Human Endothelial Cells and Macrophages
Source: Viruses. 2019 Sep 14;11(9):855. doi: 10.3390/v11090855 (PMC6784088; doi:10.3390/v11090855)
Supplement: Supplementary file 1 [file viruses-11-00855-s001.pdf]

Supplementary Data

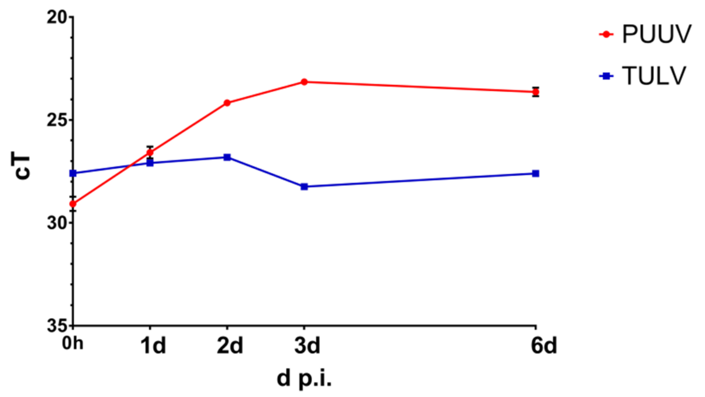

(**a**)

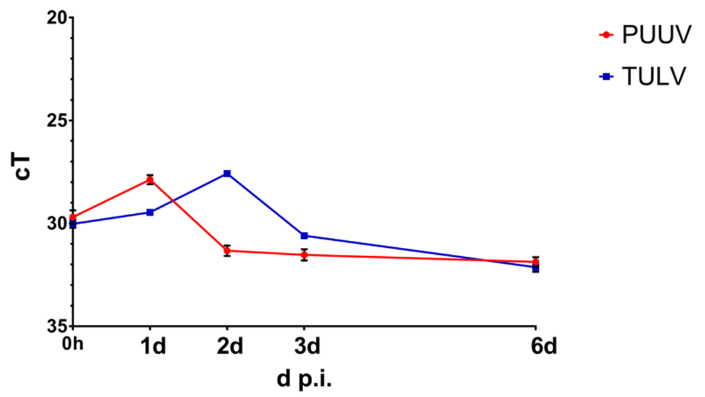

(**b**)

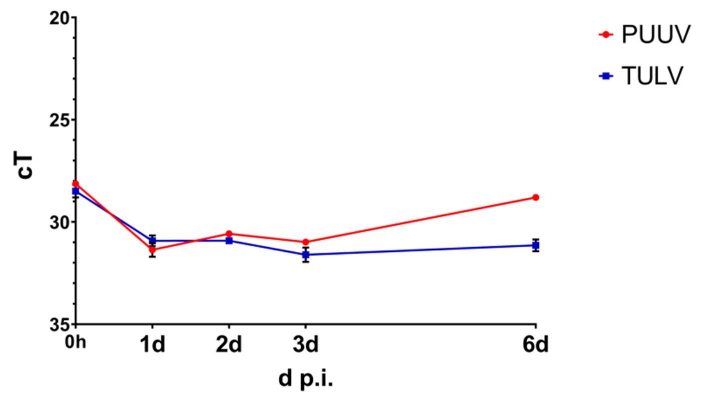

(**c**)

Figure S1. Quantification of viral RNA amplification in PUUV- and TULV-infected peripheral blood mononuclear cell (PBMC)-derived macrophages and human glomerular microvascular endothelial cells (HGMEC). The RNA replication of PUUV and TULV in a) PBMC-derived M1-like polarized and b) PBMC-derived M2-like polarized macrophages and c) HGMECs was quantified by means of

qRT-PCR analysis of viral RNA copies in cell-free cell culture supernatants. Cells were infected with a MoI of 0.1 and viral RNA copies were quantified at 0 h, 1 d, 2 d, 3 d, and 6 d post infection. All experiments were performed in duplicate using RNA samples from two independently infected cell cultures for each analysis. PBMCs were isolated from fresh human buffy coat via Ficoll-Paque density gradient cell separation. Isolation of CD14+ monocytes was done via MACS separation using CD14+ MicroBeads (Miltenyi Biotec, Bergisch Gladbach, Germany). For polarization into M1-like or M2-like macrophages, 100 ng/mL GM-CSF or 50 ng/mL M-CSF were added to the medium. HGMECs were obtained from Alphabioregen, Boston, MA, USA.

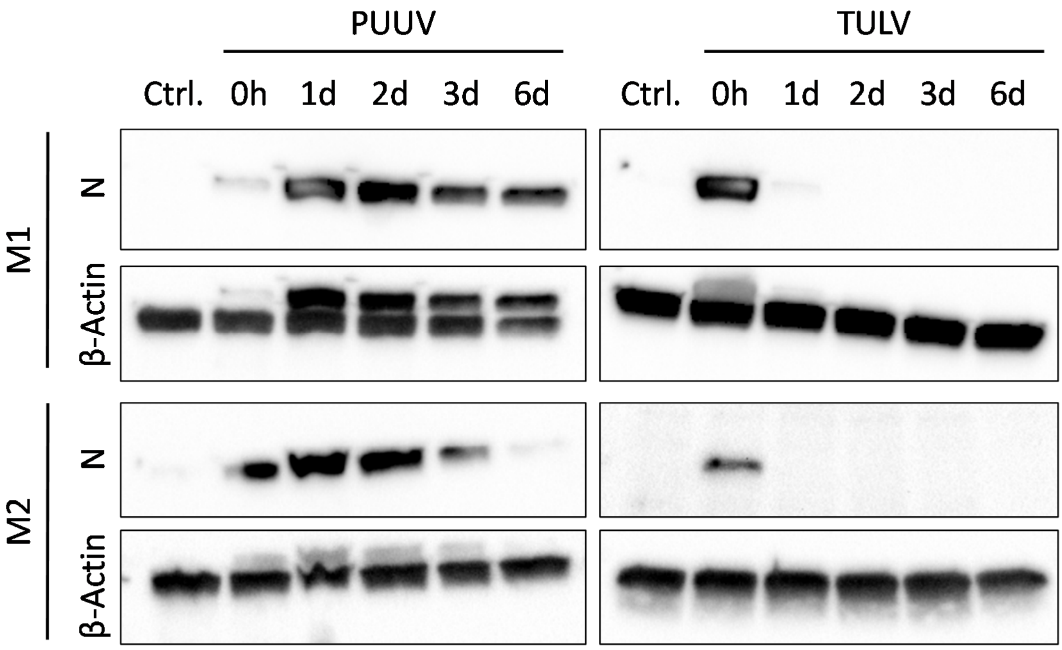

Figure S2. Nucleocaspid protein expression in PUUV- and TULV-infected PBMC-derived M1 and M2 polarized macrophages. The expression of the viral nucleocapsid (N) protein was analyzed via immunoblotting in lysates of PUUV- or TULV-infected PBMC-derived M1-like and M2-like polarized macrophages. Cells were infected with a MoI of 0.1 and N protein expression was detected at 0 h, 1 d, 2 d, 3 d, and 6 d post infection. Detection of β-Actin served as loading control.

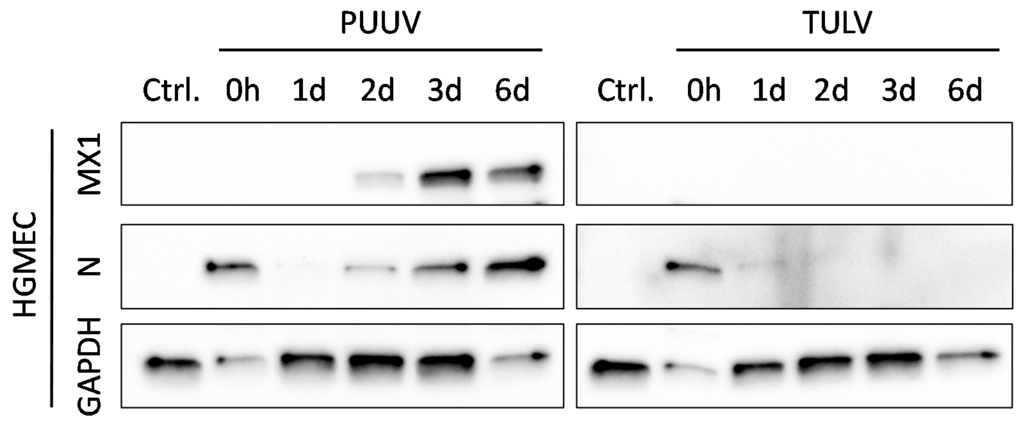

Figure S3. Nucleocaspid protein and MX1 expression in PUUV- and TULV-infected HGMECs. The expression of the viral nucleocapsid (N) protein and of the cellular MX1 protein was analyzed via immunoblotting in lysates of PUUV- or TULV-infected HGMECs. Cells were infected with a MoI of 0.1 and N protein and MX1 expression was detected at 0 h, 1 d, 2 d, 3 d, and 6 d post infection. Detection of GAPDH served as loading control.

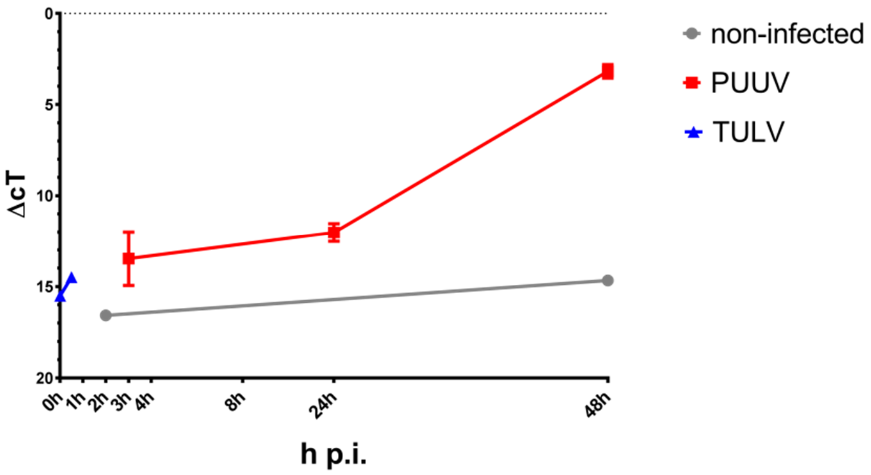

(**a**)

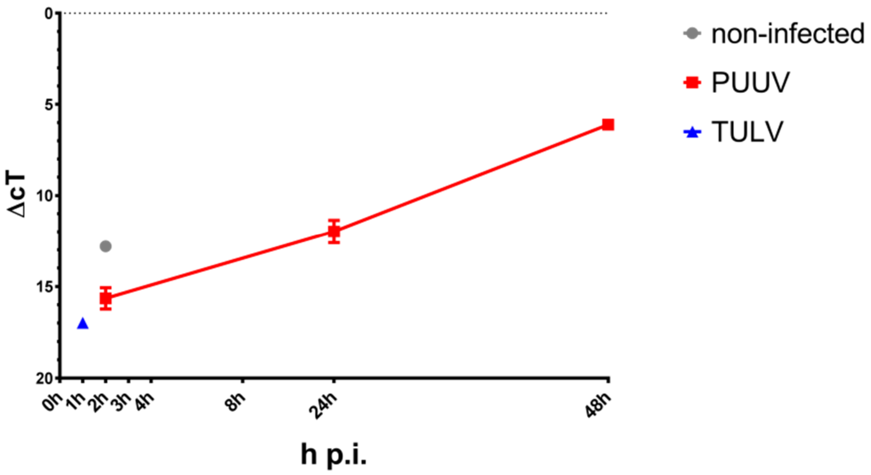

(**b**)

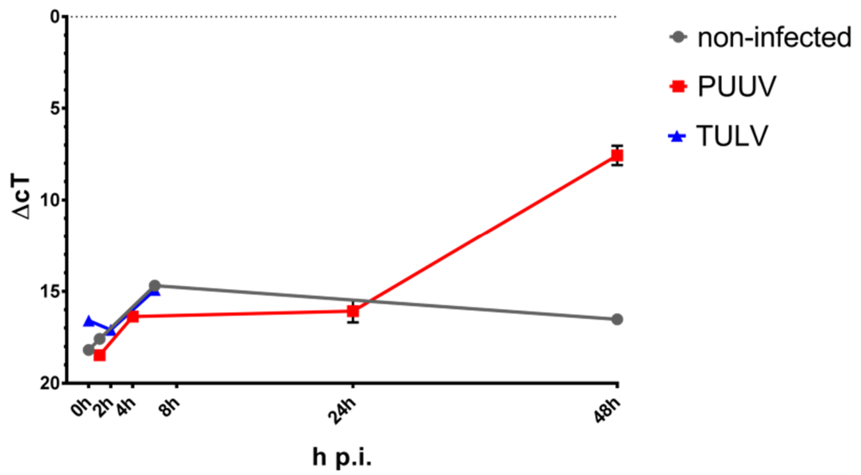

(c)

Figure S4. IFN-λ gene expression in response to PUUV or TULV infection. Expression of the *IFNL1* gene was quantified in response to PUUV or TULV infection of a) HMEC-1 or b) THP-1 cells via qRT-PCR. c) *IFNL2* gene expression was analyzed in HMEC-1 cells. Cells were infected using a MoI of 1.0 and lysed at the indicated time points, followed by extraction of total RNA and conversion to cDNA. Subsequently, the expression of the cellular *IFNL1* or *IFNL2* gene was quantified via qPCR and normalized to the expression of the cellular *MYC* gene. Missing data points indicate that no *IFNL1* or *IFNL2* gene expression was detectable.

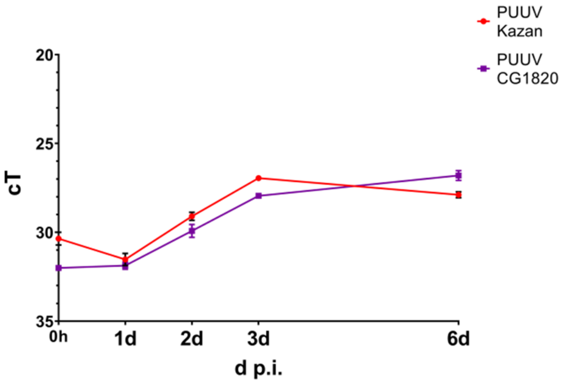

(**a**)

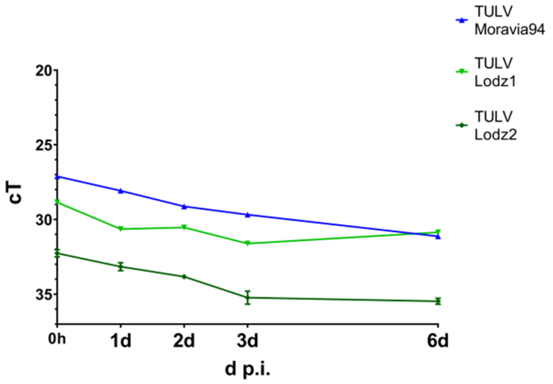

(**b**)

Figure S5. RNA replication kinetics of different PUUV and TULV strains. The RNA replication of a) PUUV strains Kazan and CG1820 and b) TULV strains Moravia and Lodz was quantified by means of qRT-PCR analysis of viral RNA copies in cell-free cell culture supernatants of infected HMEC-1 cells. Cells were infected with a MoI of 0.1 and viral RNA copies were quantified at 0 h, 1 d, 2 d, 3 d, and 6 d post infection.

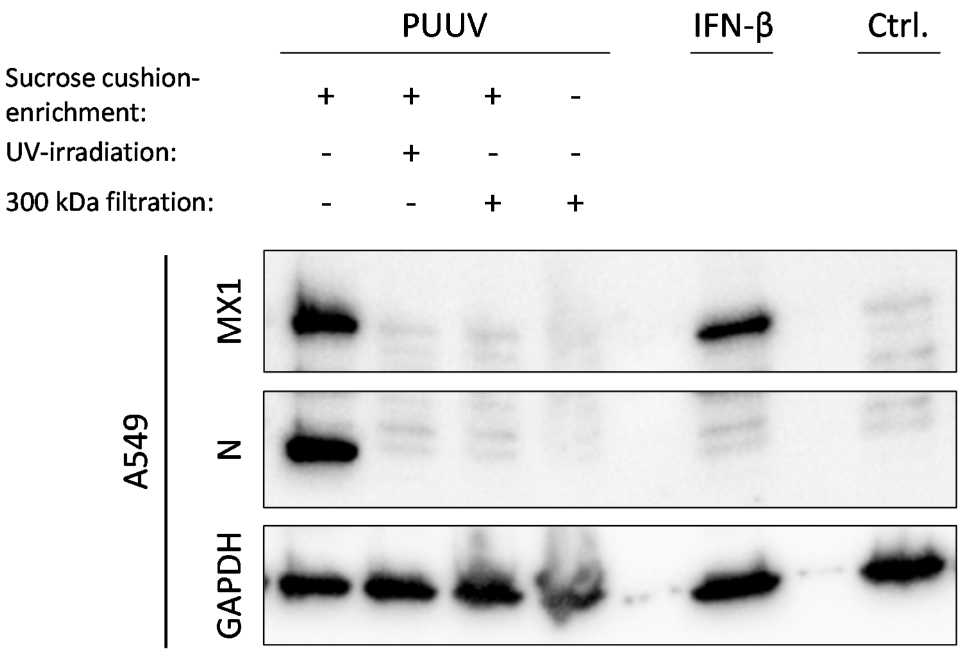

Figure S6. Nucleocapsid protein and MX1 expression in A549 cells infected with PUUV, UV-irradiated PUUV, or PUUV stocks depleted of infectious viruses. The expression of the viral nucleocapsid (N) protein and of the cellular MX1 protein was analyzed via immunoblotting in lysates of A549 cells. Cells were infected with a MoI of 1.0 and N protein and MX1 expression was detected at 3 d post infection. Detection of GAPDH served as loading control. Sucrose cushion-enriched PUUV stocks were inactivated via UV-irradiation (4 × 15 min) in a Stratalinker UV-crosslinker. Additionally, infectious viruses were removed from sucrose cushion-enriched or crude PUUV stocks via centrifugation through VivaSpin 6 300.000 MWCO exclusion filters (Sartorius, Göttingen, Germany). As a positive control, cells were treated with human IFN-β (pbl assay science, Piscataway, NJ, USA; 100 U/mL).

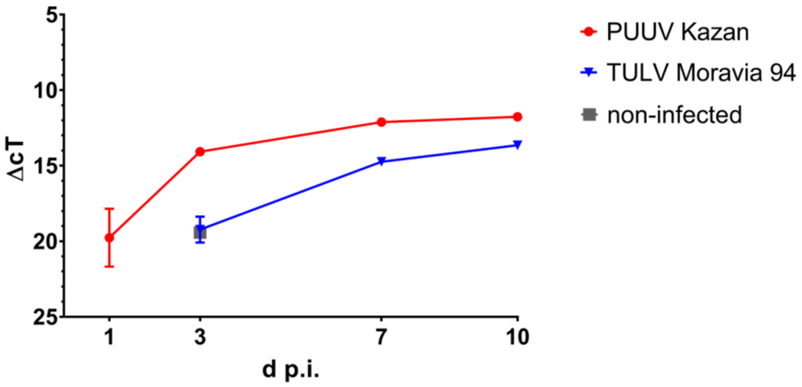

Figure S7. *IFNL1* gene expression in PUUV- or TULV-infected Vero E6 cells. Vero E6 cells were infected with PUUV or TULV at a MoI = 0.01. At 1 d, 3 d, 7 d, and 10 d p.i., total RNA was extracted from infected and mock-infected cells. *IFNL1* gene expression was quantified via qRT-PCR and normalized to expression of the cellular *MYC* gene. In the mock-infected cells, *IFNL1* gene expression was only detectable at 3 d post infection.

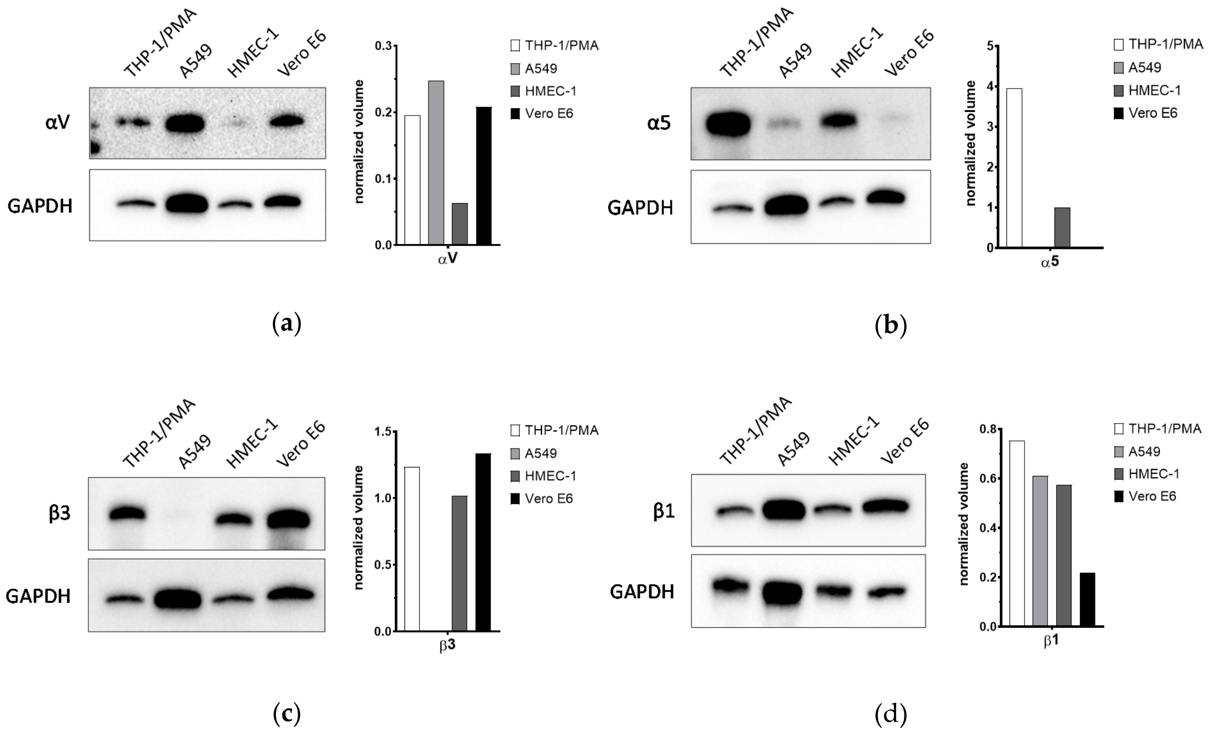

Figure S8. Integrin expression in THP-1/PMA, A549, HMEC-1, and Vero E6 cells. The expression of the cellular integrins a) αv, b) α5, c) β3, and d) β1 was analyzed via immunoblotting in lysates of the respective cells. Detection of GAPDH served as loading control. Additionally, volume quantification of individual integrin bands was done using the Image Lab software (Bio-Rad, Hercules, CA, USA), followed by normalization of signal intensities to GAPDH.

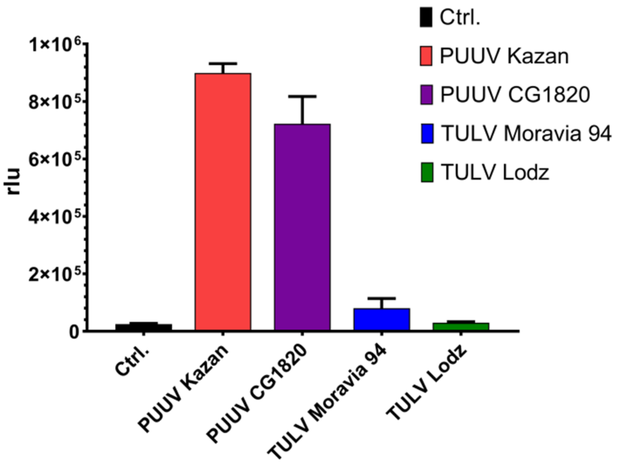

Figure S9. IRF-activity in PUUV- and TULV-infected A549-Dual™ reporter cells. The A549-Dual™ cells (Invivogen, San Diego, CA, USA) encode a secreted Lucia luciferase, which is expressed under the control of an ISG54 minimal promoter in conjunction with five IFN-stimulated response elements. The activity of the secreted Lucia Luciferase was assessed in cell culture supernatants at 6 d p.i. with either PUUV strains Kazan and CG1820 or TULV strains Moravia 94 or Lodz (MoI = 0.1) using the QUANTI-Luc™ detection reagent. Monitoring of luminescence allows to quantify the activity of the interferon regulatory factor (IRF) pathway in the cells. rlu = relative luminescence units.
